# Case report: Rapid recurrence of a chronic subdural haematoma associated with prostate cancer metastasis to a haematoma capsule

**DOI:** 10.3389/fonc.2022.988779

**Published:** 2022-08-18

**Authors:** Zhuoxuan Wu, Yi Gan, Kun Wang, Yirong Wang, Junhui Lv, Shuxu Yang

**Affiliations:** ^1^ Department of Medical Oncology, Sir Run Run Shaw Hospital, College of Medicine, Zhejiang University, Hangzhou, China; ^2^ Department of Pathology, Sir Run Run Shaw Hospital, College of Medicine, Zhejiang University, Hangzhou, China; ^3^ Department of Neurosurgery, Sir Run Run Shaw Hospital, College of Medicine, Zhejiang University, Hangzhou, China

**Keywords:** prostate cancer, chronic subdural hematoma, hematoma capsule metastasis, burr hole drainage, excision of hematomas capsule

## Abstract

**Background:**

Chronic subdural haematoma (CSDH) has various causes, including trauma, coagulopathies, and intracranial hypotension. However, CSDH associated with extracranial malignancy is rare. Here, we report an extremely rare case of CSDH due to prostate cancer metastasis to a haematoma capsule.

**Case Description:**

A 79-year-old man with a history of prostate cancer had a progressive decline in consciousness during hospitalization for cancer treatment. CSDH was diagnosed from computed tomography (CT) imaging. We urgently performed burr hole drainage, and the patient’s symptoms improved rapidly after surgery. After removing the drainage tube, the patient’s symptoms worsened again, and the repeat head CT suggested recurrence of CSDH. In a second operation, most of the haematoma capsule was excised under craniotomy, and the thickened haematoma capsule was sent for routine pathologic examination. Pathological findings confirmed the metastasis of prostate cancer to the haematoma capsule, which we believed to be related to a rapid recurrence of CSDH. After the second operation, the disease course progressed without CSDH recurrence.

**Conclusions:**

For patients with malignant tumours diagnosed with CSDH, the possibility of metastasis to a haematoma capsule needs to be considered. Burr holes and drainage can easily lead to a rapid relapse. Excision of the haematoma capsule is the key to successful treatment.

## Introduction

Chronic subdural haematoma (CSDH) is one of the most common neurosurgical diseases and usually occurs in elderly patients with a history of head trauma (1). Burr hole surgery is the mainstream treatment for CSDH, but the postoperative recurrence rate is still as high as 2.5-33% (2). Various risk factors are associated with recurrent CSDH (3–8). One of the factors is metastasis of extracranial malignant tumours, which are rare and have a devastating prognosis. Most reported cases have been associated with dural metastasis. We report an extremely rare case in which a patient with a history of prostate cancer showed rapid recurrence following the first surgical treatment for CSDH. The haematoma capsule was found to contain metastatic cells of prostate cancer, which we believe caused a rapid recurrence of CDSH.

## Case report

### Presentation

A 79-year-old man presented to our hospital with a 3-month history of shortness of breath and chest tightness. He was admitted to the cardiology department for further treatment. During this hospital stay, the patient developed progressive muscle loss of the right limb with aphasia. The cardiologist asked a neurosurgeon to evaluate the patient. The patient had a Glasgow Coma Scale (GCS) score of 10, grade 3 muscle strength of the right lower limb, and motor aphasia. The family denied that the patient had any history of head trauma. The patient had a 10-year history of prostate cancer and received radionuclide targeted therapy in another hospital before hospitalization. The patient was never treated with antiplatelet or anticoagulant drugs.

### Investigations

**Figure 1:**
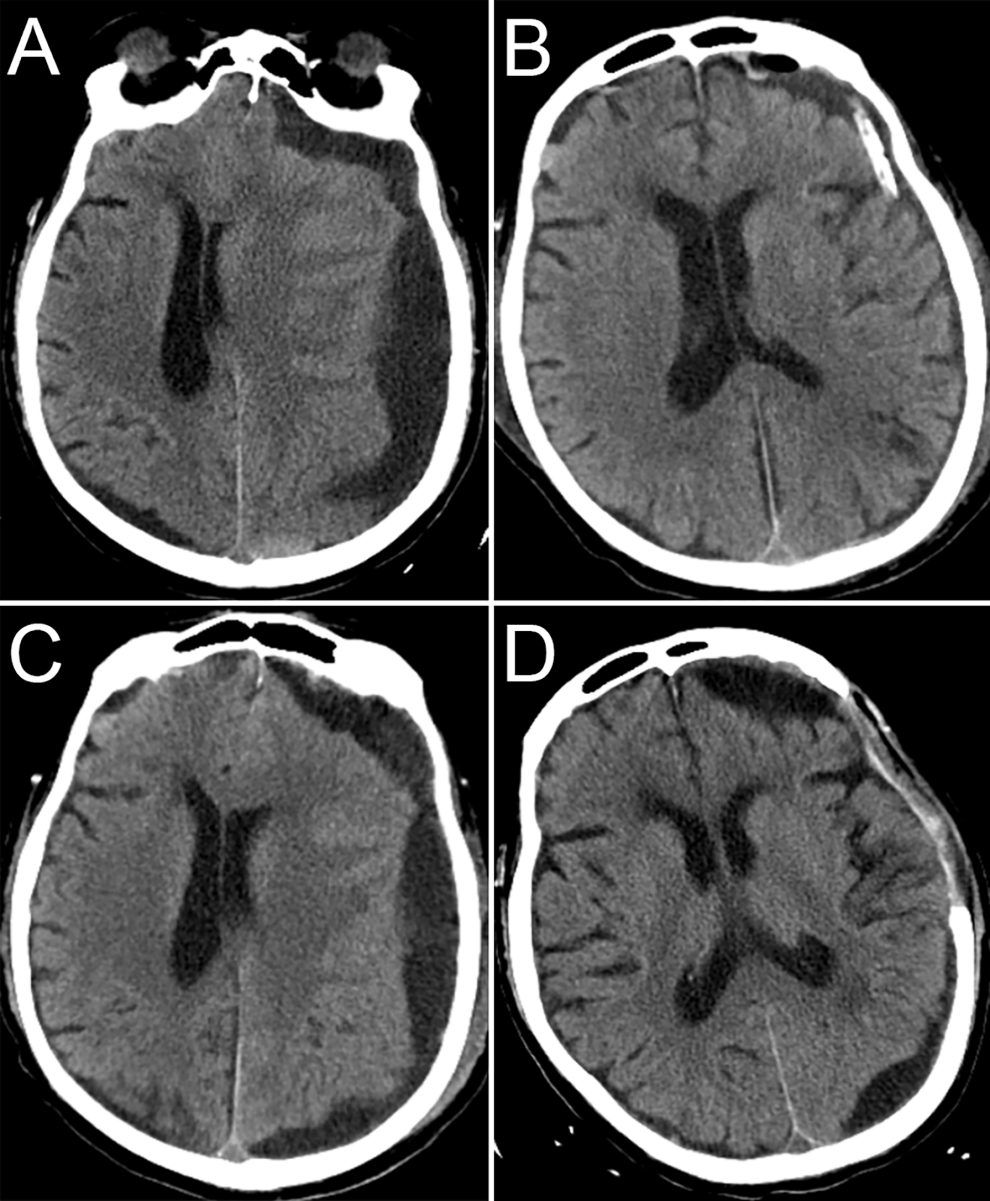
CT images during hospitalization. CT before the first surgery **(A)**, CT after the first surgery **(B)**, CT before the second surgery **(C)**, CT 1 month after the second surgery **(D)**.

Preoperative emergency cranial computed tomography (CT) demonstrated a left CSDH. The maximum thickness was 2.6 cm, and the midline structure was shifted to the right side by 1.3 cm. There was no evidence of primary craniocerebral injury or skull fracture (**Figure 1A**). The coagulation examination showed a prothrombin time of 15.4 s (reference range 11.5-14.5 s), activated partial thromboplastin time of 44.2 s (reference range 29.2-41.2 s), international standard rate of 1.25 s (reference range 0.9-1.1), and prothrombin time percent activity of 71% (reference range 80-120%). Blood routine showed platelets of 131*10^9^/L (reference range 125-350*10^9^/L).

### Management

We performed burr hole drainage for the CSDH, and dark red fluid was seen during the operation. The patient’s symptoms significantly improved after surgery. The repeat head CT indicated that the midline structure was in the middle (**Figure 1B**). The subdural drainage tube was removed 2 days after surgery. Three days after the tube was removed, the patient’s symptoms worsened again. The patient was re-examined immediately with head CT, which showed recurrence of the left subdural haematoma (**Figures 1C**). We performed emergency craniotomy to remove the haematoma, and during the operation, we found that the haematoma capsule was thickened and that the fluid was a pale red. Considering the patient’s history of malignant tumours, the haematoma capsule was excised to the maximum extent and submitted for pathological examination. We were concerned about a second recurrence of the haematoma or even cerebral hernia, and after talking to the family, the flap was removed. There was no recurrence of the chronic subdural haematoma in the follow-up (**Figure 1D**). The patient died of liver failure one month after the second surgery, not due to intracranial causes.

### Pathology

**Figure 2:**
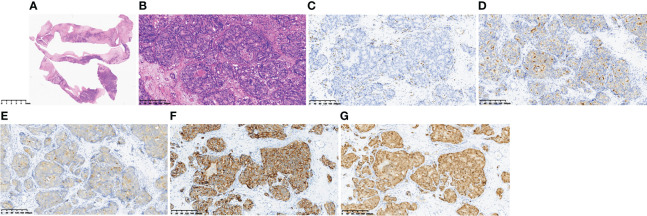
Pathological findings of the haematoma capsule. HE staining **(A, B)**, ERG immunostaining **(C),** PSA (+) **(D)**, PAP (+) **(E)**, P504S (+) **(F)**, AR (+) **(G)**.

Haematoxylin and eosin (HE) staining showed a multifocal tumour cell nest on the haematoma envelope, the nuclei were obvious and large and deeply stained, the arrangement was sieve-like or solid, and necrosis was visible in the centre (**Figures 2A, B**). ERG immunostaining showed vascular endothelium around the tumour cells. All tumour cells were located in the vascular lumen (**Figure 2C**). Staining for PSA, PAP, P504S, and AR was positive, proving that the tumour cells originated from prostate cancer (**Figures 2D–G**).

## Discussion

Malignant neoplasms are a rare cause of CSDH, leading to approximately 0.5%-4% of CSDHs (8, 9). Most cases have reported CSDHs associated with dural metastasis from malignancy. There are two other extremely rare cases. Toshiya Ichinose reported a case of CSDH cavity occupied with a metastatic tumour (10). Hiroyuki Ikeda reported another case of CSDH associated with metastasis from an extracranial malignancy to the haematoma capsule (11).

The following possible mechanisms of CSDH caused by tumours have been considered: (1) neovascularization due to dural metastasis and disruption of the neovascularization, (2) dural venous obstruction by cancer cells, causing vessel rupture or secretion of plasma components, and (3) haemorrhagic effusion due to an angiodesmoplastic reaction (12). The mechanism of CSDH recurrence due to metastasis from malignant tumours to haematoma capsules is similar to the induction of subdural haematoma through dural metastasis from malignant tumours (11). Such mechanisms include dural venous obstruction by cancer cells, which causes extravascular leakage of fluid. This may explain why our case appeared hypodense on CT before surgery and at the time of CSDH recurrence. In fact, our pathological results also confirmed that the tumour cells were all in the vascular lumen (**Figure 2C**). The other possible mechanism of CSDH recurrence is activation of the phosphoinositide 3-kinase (PI3 kinase)/Akt pathway in the outer membranes of the haematoma 13). In view of the above mechanisms, resection of the haematoma capsule may be the key to preventing postoperative recurrence.

In the present case, it is difficult to explain the reason for such a rapid recurrence other than malignant tumours. Pathological examinations revealed metastasis of prostate cancer to the haematoma capsule, which we believe may be the cause of the rapid recurrence of CSDH. We excised the haematoma capsule, and there was no repeated recurrence within 1 month after surgery, indirectly proves that the metastasis of prostate cancer to the haematoma capsule was the cause of the rapid recurrence of CSDH. Due to the size of the bone window, there is a small amount of residual haematoma capsule. There may be a risk of “acute” subdural haematoma after surgery. Postoperative CT showed a small local recurrence of the haematoma (**Figure 1D**), which indirectly confirmed our hypothesis. A limitation of this case is that we did not perform cranial contrast-enhanced magnetic resonance imaging (MRI) or dural pathology to exclude the possibility of dural metastasis.

Some tumours, including prostate tumours, are typically calcified, and involvement of the skull or dura mater is common. We tend to think of the possible relationship between calcified CSDH and the presence of metastatic tumours. The main aetiological factors associated calcified CSDHs are head trauma, shunting for hydrocephalus and subsequent cranial surgery (14, 15). A calcified CSDH often develops through a long process of formation, with a mean duration of 25.1 months (14). There is no evidence that calcified CSDH is associated with metastatic tumours, nor is it consistent with a lengthy process of formation.

Neurosurgeons should be on high alert for patients with chronic subdural haematoma complicated with malignancy. In the first operation, we ignored the possibility of chronic subdural haematoma associated with malignant tumours, and intraoperative biopsy of the dura and haematoma capsule was not performed. If we had a better understanding of these diseases and had performed rapid frozen pathology to confirm metastasis, we would have expanded the bone window craniotomy and removed the haematoma capsule, avoiding the recurrence of CSDH. However, considering the mechanism of CSDH recurrence and the case of failure (11), middle meningeal artery embolization is not suitable for these patients.

## Conclusions

Metastasis of an extracranial malignancy to the CSDH capsule is extremely rare, and we believe that was the cause of the rapid recurrence of CSDH. For patients with chronic subdural haematoma complicated with malignant tumours, we need to be highly aware of the possibility of capsule metastasis, and rapid frozen pathological examination should be conducted during surgery in such cases. Excision of the haematoma capsule is the key to surgical treatment.

## Data availability statement

The original contributions presented in the study are included in the article/supplementary material. Further inquiries can be directed to the corresponding authors.

## Ethics statement

Written informed consent was obtained from the individual(s) for the publication of any potentially identifiable images or data included in this article.

## Author contributions

Conceptualization: SY and JL. Investigation and data collection: ZW. Pathological diagnosis: YG. Surgery: SY, JL, KW, and YW. Writing - original draft: ZW and JL. Writing - review & editing: SY. All authors reviewed and approved the final manuscript.

## Funding

This research was supported by Zhejiang Provincial Natural Science Foundation of China under Grant No. LQ20H160042.

## Conflict of interest

The authors declare that the research was conducted in the absence of any commercial or financial relationships that could be construed as a potential conflict of interest.

## Publisher’s note

All claims expressed in this article are solely those of the authors and do not necessarily represent those of their affiliated organizations, or those of the publisher, the editors and the reviewers. Any product that may be evaluated in this article, or claim that may be made by its manufacturer, is not guaranteed or endorsed by the publisher.
